# Caffeine and Its Antioxidant Properties—It Is All about Dose and Source

**DOI:** 10.3390/ijms232113074

**Published:** 2022-10-28

**Authors:** Bianca-Eugenia Ősz, George Jîtcă, Ruxandra-Emilia Ștefănescu, Amalia Pușcaș, Amelia Tero-Vescan, Camil-Eugen Vari

**Affiliations:** 1 Department of Pharmacology and Clinical Pharmacy, Faculty of Pharmacy, George Emil Palade University of Medicine, Pharmacy, Science and Technology of Targu Mures, 540139 Targu Mures, Romania; 2 Department of Pharmacognosy and Phytotherapy, Faculty of Pharmacy, George Emil Palade University of Medicine, Pharmacy, Science and Technology of Targu Mures, 540139 Targu Mures, Romania; 3 Department of Biochemistry and Chemistry of Environmental Factors, Faculty of Pharmacy, George Emil Palade University of Medicine, Pharmacy, Science and Technology of Targu Mures, 540139 Targu Mures, Romania; 4 Department of Biochemistry, Faculty of Medicine, George Emil Palade University of Medicine, Pharmacy, Science and Technology of Targu Mures, 540139 Targu Mures, Romania

**Keywords:** caffeine, oxidative stress, coffee, cocoa, sweets, xanthine oxidase

## Abstract

Caffeine is the most frequently used substance with a central nervous system stimulant effect, but its consumption is most often due to the intake of foods and drinks that contain it (coffee, tea, chocolate, food supplements with plant extracts of Guarana, *Mate herba*, Cola nuts). Due to its innocuity, caffeine is a safe xanthine alkaloid for human consumption in a wide range of doses, being used for its central nervous stimulating effect, lipolytic and diuresis-enhancing properties, but also as a permitted ergogenic compound in athletes. In addition to the mechanisms that explain the effects of caffeine on the targeted organ, there are many proposed mechanisms by which this substance would have antioxidant effects. As such, its consumption prevents the occurrence/progression of certain neurodegenerative diseases as well as other medical conditions associated with increased levels of reactive oxygen or nitrogen species. However, most studies that have assessed the beneficial effects of caffeine have used pure caffeine. The question, therefore, arises whether the daily intake of caffeine from food or drink has similar benefits, considering that in foods or drinks with a high caffeine content, there are other substances that could interfere with this action, either by potentiating or decreasing its antioxidant capacity. Natural sources of caffeine often combine plant polyphenols (phenol-carboxylic acids, catechins) with known antioxidant effects; however, stimulant drinks and dietary supplements often contain sugars or artificial sweeteners that can significantly reduce the effects of caffeine on oxidative stress. The objective of this review is to clarify the effects of caffeine in modulating oxidative stress and assess these benefits, considering the source and the dose administered.

## 1. Introduction

Caffeine is the most used substance with a centrally excitatory effect since it is a common ingredient in beverages and foods. According to data published by the EFSA (European Food Safety Authority), the average daily consumption of caffeine in young adults (18–65 years old) is 37–319 mg and derives mainly from products based on coffee and cocoa beans, tea leaves, guarana berries, and kola nuts [1]. The consumption of caffeine begins in childhood and resides in sweets and juices, but in conditions of moderate consumption, the doses are low and do not raise health concerns [2]. After oral administration, caffeine is rapidly absorbed with a bioavailability of approximately 99%. It is distributed in most tissues, including the brain, and then it is metabolized in the liver and excreted via the kidneys, mostly in the form of metabolites [3].

Although caffeine has been commonly used since ancient times, it is still a studied and controversial substance. The studies published in the literature regarding this substance convey both the beneficial effects and toxic effects (dose-related) on health. In recent years, however, several studies have attempted to establish the link between caffeine and oxidative status, as many of the conditions in which caffeine has been shown to have a beneficial influence can be attributed to a decrease in oxidative stress (OS).

Oxidative stress is defined as an imbalance between the production of reactive species and the ability of extracellular and intracellular protection systems to neutralize them. A growing number of medical conditions are correlated with the increase in the level of reactive oxygen species (ROS), but the pathophysiological mechanisms in which they are involved are beyond the purpose of our paper, and, in addition, their positive effects should not be excluded [4]. Briefly, these species originate from the oxygen molecule (O_2_), which in the presence of a free electron, forms the superoxide anion ($O_{2}^{*-}$). The latter, under the action of superoxide dismutase (SOD), is converted to hydrogen peroxide (H_2_O_2_), a more stable compound with a half-life longer than $O_{2}^{*-}$. However, both $O_{2}^{*-}$ and H_2_O_2_, in the presence of transitional metals (Cu, Fe, Zn), generate a highly reactive free radical, the hydroxyl radical (•OH), through the Fenton and Haber–Weiss reactions. In addition, $O_{2}^{*-}$ can interact with nitric oxide (NO), forming reactive nitrogen species (RNS), such as peroxynitrite (ONOO^−^), from which •OH can be obtained [5,6,7].

## 2. Pharmacokinetic and Pharmacodynamic Properties of Caffeine

### 2.1. Caffeine: Mechanism of Action

The peripheral and central effects of caffeine in the currently used doses occur as a result of antagonizing P1 adenosine receptors [8]. P1 receptors have four subtypes A1, A2A_,_ A2B, and A3. Xanthine derivatives, such as caffeine, theophylline, and other naturally occurring xanthines, have an affinity for all four receptor subtypes. A1 and A3 receptors are negatively coupled to adenylate cyclase (AC) and decrease cyclic adenosine monophosphate (cAMP) concentration, while A2A and A2B receptors stimulate AC activity and increase intracellular cAMP levels [9]. Since the localization of these receptors is different, the consequent effects of their stimulation or blocking are complex. For example, the density of A1 receptors is increased in the heart so that antagonists of these receptors, such as caffeine, stimulate the heart. The A2A subtype is predominantly found in coronary arteries; as a consequence, adenosine has coronary dilator effects. [10].

Adenosine receptors are also located in the kidney and are involved in the control of the glomerular filtration rate (GFR) and the hydro-electrolytic balance. More specifically, through A1 receptors, adenosine produces afferent arteriolar constriction and decreases GFR, which implies that antagonists of these receptors increase GFR and diuresis, specifically natriuresis [11].

In the CNS, adenosine favors the release of neurotransmitters through A2A receptors and inhibits it through A1 receptors, modulating sleep and vigilance [12]. The central stimulating action of caffeine is explained by antagonizing adenosine receptors [13]. A2A agonists have been shown to have pro-nociceptive effects, the intensity of the pain being directly proportional to the increase in cAMP level [14]. Moreover, the stimulation of A2A receptors located in the pial vessel causes vasodilatation [15], which can contribute to the occurrence of headaches and migraines. Therefore, the use of caffeine in migraine treatment is due to antagonizing these effects [14].

Caffeine is a non-selective inhibitor of phosphodiesterase (PDE) enzymes involved in the inactivation of cAMP and cyclic guanosine monophosphate (cGMP) with the formation of 5′-AMP and 5′-GMP. This increases the level of cAMP and cGMP [16]. Mammalian PDEs, divided into 11 subfamilies (PDE1 to PDE11), have different specificities for cyclic nucleotides. PDEs 4, 7, and 8 selectively hydrolyze cAMP; PDEs 5, 6, and 9 selectively hydrolyze cGMP; and PDEs 1, 2, 3, 10, and 11 possess an affinity for both cAMP and cGMP. The tissue distribution of these isoforms is variable, and modulating their activity has different consequences [17]. The effects of caffeine that can be explained as a result of the inhibition of PDEs include the (weak) bronchodilator effect and the lipolytic effect [8].

**Figure 1:**
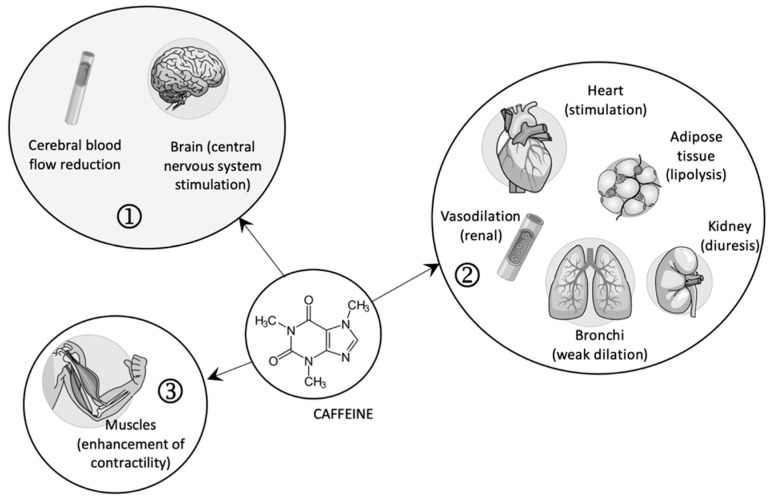
The pharmacological effects of caffeine (1—central effects; 2—peripheral effects; 3—contraction of skeletal muscles).

In high doses, caffeine, as an agonist of ryanodine receptors, favors the contraction of striated muscles by releasing calcium ions from the sarcoplasmic reticulum (see Figure 1) [18].

Although the complex effects of caffeine are explained in specialized literature based on the three mechanisms presented above, the effects are different depending on the dose used for the antagonism of adenosine receptors, the inhibition of PDE enzymes, and the mobilization of calcium from the endoplasmic reticulum. For example, the affinity for adenosine receptors was observed only in low doses and not high ones [13,19], while the consequent effects of PDEs inhibition are especially visible in doses several times higher than those currently used due to the low affinity of caffeine on PDEs [13].

Since these effects of caffeine are well known and intensively discussed in the literature, only the mechanisms involved in the modulation of oxidative stress will be detailed in this paper.

Although the beneficial effect of caffeine to reduce OS is well documented and demonstrated in both in vivo and in vitro studies, the way it does so has not been fully elucidated.

### 2.2. Caffeine: The Metabolic Pathways Involved in Biotransformation and Oxidative Stress

**Figure 2:**
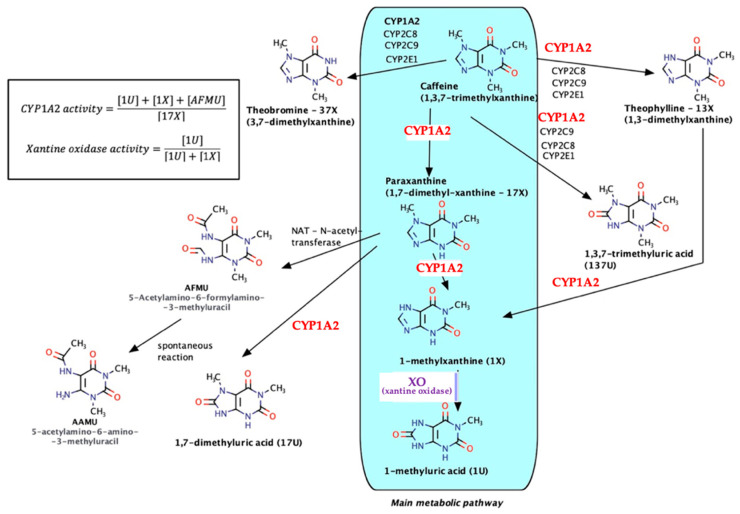
Caffeine metabolic pathways.

As a natural xanthine derivative, structurally similar to endogenous purine nitrogenous bases, caffeine is metabolized almost entirely and involves both microsomal (especially CYP1A2) and non-microsomal enzyme xanthine oxidase (XO)—see Figure 2.

The main metabolic pathway is the formation of paraxanthine (17-X) followed by N7-demethylation, reactions via CYP1A2 (80% of the total amount), and then oxidation under the action of XO with the formation of 1-methyluric acid (1U). Secondary metabolites (theobromine, theophylline, trimethyluric acid, etc.) each occur in much lower percentages (6–8%). As a result, taking into account the relative innocuity of caffeine, the urinary metabolites/caffeine can be used in pharmacogenetics for the determination of the CYP1A2 phenotype (molar ratio of 7-demethylated compounds–AFMU, 1-methylxanthine, 1-methyluric acid, and the immediate precursor paraxanthine), as for the quantitative evaluation of the catalytic activity of XO (molar ratio of 1-methyluric acid to total 1-methyluric acid + 1-methylxanthine [20,21,22,23,24].

As a substrate of XO, caffeine and its metabolites can competitively inhibit uric acid formation. The latter compound has dual characteristics—at the extracellular level, it acts as a powerful antioxidant, but inside the cell, it acts as a promoter of the formation of ROS; at the intracellular level, uric acid is a compound that generates endothelial dysfunction by favoring the formation of cytokines and lipid peroxidation, reducing the amount of endothelial nitric oxide, and favoring the pro-inflammatory cascade ERK/p38 MAPK [25,26].

Mammalian XO comes from hepatic xanthine dehydrogenase (XDR), which has an oxidizing action on some biological substrates via NAD^+^ with the consequent formation of NADH; after its release into plasma, XDR, following partial proteolysis, will produce XO which, concomitantly with the formation of uric acid, generates the formation of ROS [27,28].

In many pathological processes in which OS has an essential role, increased XO activity is involved: type 1 diabetes mellitus, in which the production of mitochondrial superoxide is not modified, and the generation of ROS thus becomes XO-dependent [29], myocardial ischemia and reperfusion lesions [30], atherosclerosis [25], and neoplastic processes [31].

Recent studies point out that longevity itself is negatively correlated with the level of OS, although antioxidant medication brings only modest benefits, except for ischemia-reperfusion syndrome in which scavenger and trapping drugs are ineffective, while the inhibition of XO can be life saving [31]. A recent retrospective clinical cohort study in the Irish population by Ruiz F et al. confirmed that hyperuricemia is an independent marker of mortality in both sexes [32].

The effects of caffeine on uricemia are still controversial. As a result of the structure and metabolites that form, caffeine is a substrate for XO, competitively interfering with purine endogenous compounds that represent the physiological substrate of the enzyme (xanthine, hypoxanthine). However, the effects of exogenous caffeine intake on uric acid levels remain questionable. Conversely, if the caffeine intake comes from natural sources (tea, coffee), the present phytocompounds may also be XO inhibitors (flavonoids, polyphenols). Thus, in an in vitro study on human immortalized normal liver cell line HL-7702, Wu D et al. showed that tea and its components inhibited XO and decreased the mRNA expression of XDR, although this effect was not attributed to caffeine but to catechins and gallic acid [33]. A meta-analysis conducted by Peluso I et al. on the effect of tea extracts in patients with asymptomatic hyperuricemia did not reach a clear conclusion due to the lack of bioavailability data of flavanol-type compounds and the small number of randomized studies used in the statistical analysis [34]. Another meta-analysis based on nine clinical studies demonstrated that daily coffee intake decreases the plasma level of uric acid, the effect being gender specific (more obvious in men than in women despite an equivalent dose) [35]. A clinical study conducted on the Korean population aimed at assessing the consumption of pure caffeine and beverages containing caffeine (tea, coffee) on the catabolism of endogenous purines and serum uric acid level; the authors concluded that regardless of the source, the moderate intake of caffeine does not influence the plasma level of uric acid in any way [36]. The contradictory results obtained by various researchers (decrease, increase, or lack of influence on uricemia) may be due to the difference in the source of caffeine (pure caffeine, extracts of tea, coffee, *Mathe folium*, guarana, etc.), but also by the polymorphism of CYP1A2 and XO. The variable activity of the two enzymes could explain gender and racial differences in caffeine metabolism with consequences in uric acid production [37]. Previous studies showed a higher CYP1A2 activity in men than in women, in Europeans than in South Africans, as well as in Swedes than in Koreans [38]. Other phytochemical compounds (catechins, flavonoids, etc.) present in natural extracts can influence the biological effect through multiple mechanisms (antioxidant effect, inhibition of XO).

### 2.3. The Main Mechanisms of Action of Caffeine in Correlation with Oxidative Stress

#### 2.3.1. Caffeine and the Purinergic Signaling Pathway

The purinergic signaling pathway is mediated by extracellular adenosine, adenosine diphosphate (ADP), and adenosine triphosphate (ATP). Nucleotides and nucleosides are purinergic receptor agonists. Nucleotides stimulate the P2X (receptor-gated ion channels) and P2Y (G protein-coupled receptors) receptors, while adenosine is a P1 receptor agonist (P1R-G-coupled receptors) [39].

If we refer to the effects of caffeine in relation to the type of receptor antagonized, the effects are dependent on the type of consumption. Although caffeine has an affinity for both A2A and A1 receptors, in the case of acute administration, the effects are specific to blocking A1 receptors. In the case of chronic consumption, however, the establishment of tolerance of A1 receptors to caffeine was observed, and the effects were specific to A2A receptor antagonism [40]. Taking into account the fact that the stimulation of both receptor subtypes changes the concentration of cAMP, with a decrease in cAMP in the case of the stimulation of A1 receptors and an increase in cAMP in the case of the stimulation of A2A receptors [9], the question arises whether the antioxidant/beneficial action of caffeine could be due to this second messenger.

The way cAMP modulates OS has not been elucidated. The decrease in cAMP in vascular smooth muscle cells was correlated with an increase in the production of the $O_{2}^{*-}$ superoxide anion [41]. In the central nervous system, cAMP-activated protein kinase A (PKA) forms at the mitochondrial level a complex with A-kinase anchoring protein 121 (AKAP121), which phosphorylates the fission modulator dynamin-related protein 1 (Drp1) with the increase in the level of reduced glutathione (GSH), but also the activity of mitochondrial SOD2. Thus, cell apoptosis is prevented after exposure to neurotoxic substances [42]. cGMP has beneficial effects, such as protecting cells against redox stress by activating antioxidant gene expression and against chromosomal instability [43,44]. As far as the second action mechanism of caffeine is concerned, PDE inhibition (in high doses) would have the effects described above.

Even if the antioxidant effects of caffeine consumption could not be directly correlated with cAMP and/or cGMP levels, these effects have been demonstrated in experimental studies.

*The importance of purinergic receptors in neurodegenerative disorders*

In a study by Leite et al., caffeine and another A2A purinergic receptor antagonist were shown to have similar effects on memory impairment as assessed using the Novel Object Recognition (NOR) and OS generated by aging tests in rats. Administration of both compounds led to a decrease in ROS and RNS, which suggested that the beneficial effect was correlated with the blocking of these receptors [45]. Neurodegeneration and the accumulation of beta-amyloid plaques (amyloid-beta accumulation) (Aβ) from Alzheimer’s disease (AD) have also been shown to be correlated with the release of inflammatory cytokines following A2A receptor stimulation. The beneficial effect of caffeine in reducing memory impairment by regulating the expression of brain-derived neurotrophic factor (BDNF) and tropomyosin receptor kinase B (TrkB) or by regulating the expression of nuclear erythroid 2-related factor (Nrf2) and Toll-like receptor 4 (TLR 4)-induced glial cells-mediated neuronal cell death and neuroinflammation [46].

Due to the complex interrelations between adenosine and nucleotides, the role of adenosine in the pathogenesis of neurodegenerative diseases cannot be separated from its phosphorylate derivatives.

The role of purinergic receptors in the pathogenesis of some degenerative CNS diseases has been intensively studied, and the effects following the stimulation and the blocking of these receptors are extremely complex. For example, OS has been observed to inhibit hydrolases (ecto-nucleotidase, E-NTPDase1/CD39 and ecto-5′-nucleotide/CD73) involved in the conversion of ATP into ADP, ADP into AMP, and AMP into adenosine, thus disrupting the redox homeostasis of the cell. This is because adenosine, via the purinergic P1 receptors, increases the activity of intracellular antioxidant systems, thereby decreasing the production of ROS. Once the activity of these ecto-enzymes is inhibited, the activation of adenosine-controlled antioxidant systems is prevented [39].

In AD, once Aβ-plates accumulate, ATP release increases in the extracellular space that activates the P2X7 receptors. In the CNS, this translates into an increase in the release of cytokines and chemokines and a decrease in α-secretase (proteolytic enzymes that cleave the amyloid precursor). However, the stimulation of P2Y2 receptors has the opposite effect, as it stimulates α-secretase activity with neuroprotective effects. On the other hand, stimulation of the P2Y13 receptors by ADP increases the activity of heme oxygenase-1 (HO-1), an enzyme involved in cytoprotection [47]. The role of P2Y13 receptors in the reduction of OS generated by H_2_O_2_ has also been demonstrated on N2A neuroblastoma cells, and it has been observed that the agonists of these receptors activate HO-1. The HO-1 activation is, however, dependent on the presence of the transcription factor Nrf2 [48].

Based on the above, it can be concluded that the harmful effects following the activation of P2Y7 receptors are counteracted by the activation of P2Y2 receptors, P2Y13 receptors, and A2A receptors.

In the pathogenesis of Parkinson’s disease (PD), on the other hand, P2Y6 receptors appear to be involved, and their stimulation causes an increase in cytokine production. This has been demonstrated in vitro on neuronal SH-SY5Y cells (cell cultures used in the study of the molecular and cellular mechanisms of toxic substances contributing to the development of PD), whereby exposure to 1-methyl-4-phenylpyridinium (MPP+), a compound with toxicity to dopaminergic neurons, increases the density of P2Y6 whereas the administration of a receptor agonist enhances its toxicity. At the same time, in the case of the knockdown of P2Y6R cells or following the use of an antagonist of these receptors, the harmful effects of MPP+ are reduced, which is demonstrated by a decrease in the production of ROS [49].

**Figure 3:**
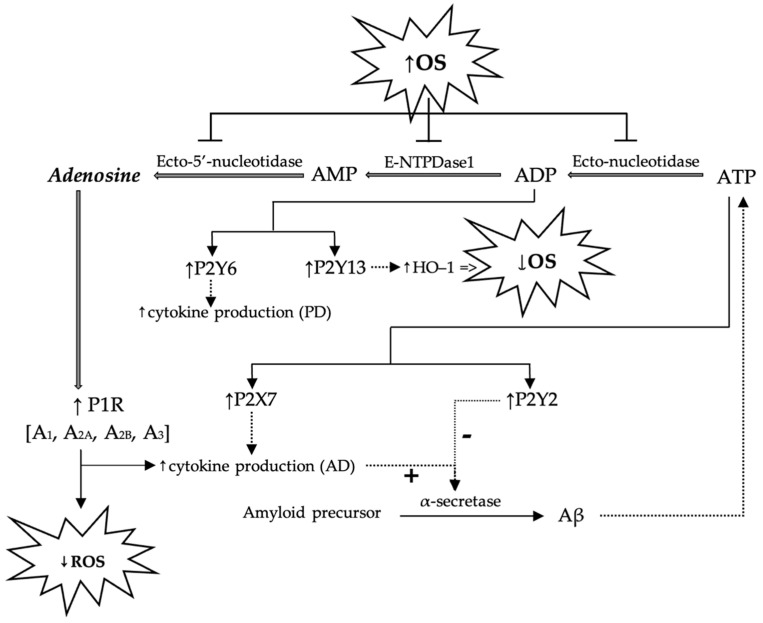
Schematic presentation of possible mechanisms by which adenosine and purine nucleotides (AMP, ADP, ATP) modulate ROS generation and possible implications in the pathogenesis of Alzheimer’s and Parkinson’s diseases. P1R—purinergic receptor 1; OS—oxidative stress; ROS—reactive oxygen species; Aβ—beta-amyloid; AD—Alzheimer’s disease; PD—Parkinson’s disease.

The role of purinergic receptors in the pathogenesis of Alzheimer’s and Parkinson’s diseases, as well as the interrelationships proposed in the literature between purinergic signaling pathways and OS, are schematically presented in Figure 3.

The role of P2X7 receptors in the modulation of autophagy and inflammation has also been studied in a nonalcoholic steatohepatitis (NASH) model, the authors noting that P2X7 receptor-deleted mice were protected in developing liver inflammation and fibrosis [47].

On the other hand, in vitro and in vivo studies have shown that caffeine potentiates SOD2 activity by inhibiting its inactivation by acetylation. This is possible due to its affinity for sirtuin 3 (SIRT3), a mitochondrial nicotinamide adenine dinucleotide (NAD^+^)-dependent on acetylase, involved in the deacetylation process of two lysine residues (lysine 68 and 122) of SOD. The binding of caffeine to SIRT3 increases the affinity of deacetylase for the substrate with the increased antioxidant capacity of SOD [50].

The destruction of cholinergic neurons in Alzheimer’s disease and dopaminergic neurons in Parkinson’s disease has been correlated with high levels of ROS and neuroinflammation. Caffeine has often been proposed as an antioxidant agent with beneficial effects in the management of neurodegenerative diseases. Among the proposed mechanisms is the enhancement of the Nrf2 effect and its associated genes and the blocking of A2A receptors [46]. Additionally, in an animal model at menopause (ovariectomized female rats), caffeine has shown a beneficial effect in reducing the anxiety of this period, with the effects demonstrated by a behavioral study (elevated plus maze test). In this study, the effectiveness of caffeine was also explained by its ability to reduce lipid peroxidation and improve the ability of antioxidant systems in the brain to reduce the level of ROS [51].

**Figure 4:**
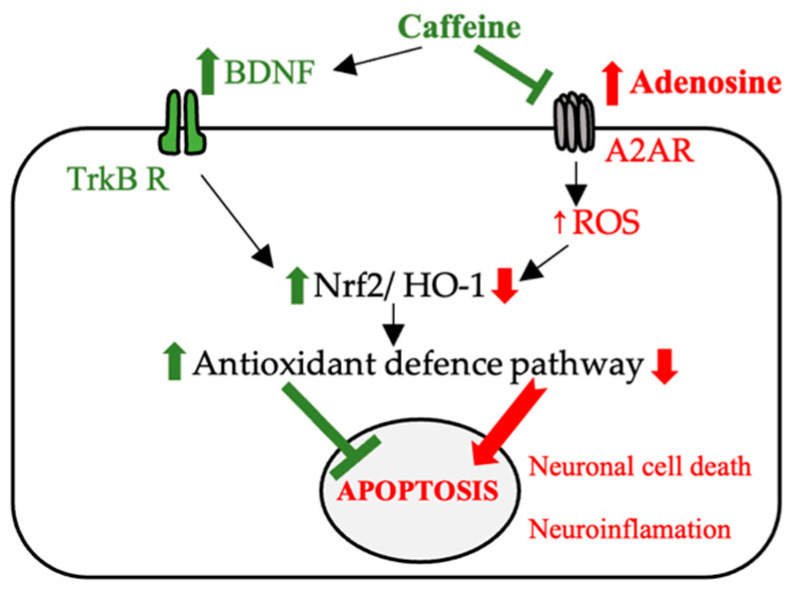
The possible mechanisms involved in the neuroprotective effect of caffeine (BDNF—brain-derived neurotrophic factor, TrKB R—tropomyosin receptor kinase B, Nrf2—nuclear erythroid 2-related factor, A2AR—adenosine receptor, HO-1—heme oxygenase-1, ROS—reactive oxygen species).

The suggested mechanisms with which caffeine has neuroprotective effects (e.g., AD and PD) are reshown in Figure 4.

In preclinical studies, caffeine has demonstrated a protective effect against the occurrence of neural damage produced after administration of d-galactose [52], cadmium (Cd) [53], lipopolysaccharides (LPS) [54], or exposure to hyperoxia [55]. The doses of pure caffeine used in these studies were 3 mg/kg/day ip (against d-galactose injuries) [52], 30 mg/kg/day ip (against Cd and LPS injuries) [53,54], and 10 mg/kg/day ip (against hyperoxia injuries) [55]. If the harmful effects of exposure to d-galactose, Cd, or LPS have been studied in adult animals, the animal model of hyperoxia has been used to assess the neuroprotective effects of caffeine at critical periods of brain development in 6-day-old rats. The brain of rats at six days post-partum corresponds, from the point of view of development to that of the human fetus in the 28–32 weeks of gestation, and this animal model can be used to evaluate the sequelae of prematurity triggered by OS and free radical-mediated tissue damage [55].

The neuroprotective effect of caffeine after exposure to neurotoxic compounds has been demonstrated in numerous in-vivo preclinical studies. The results are summarized in Table 1.

In patients with Parkinson‘s disease, consumption of caffeine for 6 weeks in doses of at least 200 mg/day was associated with an improvement in motor symptoms, and a slowing of the progression of dyskinesia was observed [60,61]. Based on these observations, the benefits of caffeine were evaluated in a placebo-controlled phase 3 trial, with no improvement in motor symptoms observed after 6 months of daily consumption of 400 mg of caffeine [62]. Therefore, even if preclinical studies have shown a neuroprotective effect of caffeine in PD patients, the results of clinical studies are controversial, the results being dependent on the stage of the disease, chronic drug treatment, duration of treatment, and dose of caffeine.

However, epidemiological studies and meta-analyses reinforce the idea of a direct correlation between caffeine/coffee consumption and decreased risk/incidence of neurodegenerative diseases, as shown in Table 2.

*The importance of purinergic receptors for liver function*

**Figure 5:**
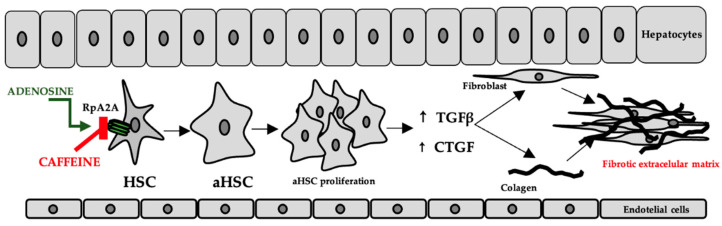
The role of adenosine and caffeine in the occurrence/prevention of liver fibrosis (HSC—hepatic stellate cells, aHSC—activated hepatic stellate cells, TGFβ—transforming growth factor beta, CTGF—connective tissue growth factor).

In the liver, adenosine is released either as a result of hypoxia or as a result of exposure to hepatotoxic compounds. The occurrence of liver fibrosis following chronic alcohol consumption has been explained by increased adenosine levels and the consequent stimulation of A2A receptors on the surface of hepatic stellate cells [69]. This has been correlated with the increased expression of transforming growth factor beta (TGFβ) and connective tissue growth factor (CTGF), which contribute to fibroblast proliferation, stimulation of collagen, and extracellular matrix synthesis [70], while purinergic receptor antagonists have been shown to have an effect on reducing these consequences [69], the proposed mechanisms include both the decrease in inflammatory biomarkers and oxidative stress [71]. The mechanism is shown in Figure 5.

Preclinical studies conducted on rats showed that the administration of caffeine in doses of 30 mg/kg/day and 100 mg/kg/day has beneficial effects on oxidative stress leading to a decrease in MDA values (in a dose-dependent manner) and advanced oxidation protein products (AOPP). The decrease in the AOPP level reflects the ability of cells to protect themselves against OS injury [72]. Caffeine has also been shown to be effective in improving biological markers in models of liver damage. Thus, administration of 37.5 mg/kg per day significantly reduced plasma levels of tumor necrosis factor-α (TNF-α), interleukin-1β (IL-1β), and interleukin-6 (IL-6), decreased malondialdehyde (MDA) and increased GSH and hepatic glutathione peroxidase (GPx) in male rats with thioacetamide-induced liver disease. In addition to improving biochemical markers, caffeine has also improved the histological and functional appearance of the liver [71]. The benefits of caffeine consumption in reducing liver bone were also evaluated in an animal model of nonalcoholic fatty liver disease induced by a high-fat diet. In this case, animals treated with caffeine at 20 or 30 mg/kg for 8 weeks had lower plasma levels of alanine aminotransferase (ALT), aspartate aminotransferase (AST) and bilirubin, and increased the level of low albumin. The authors also assessed how caffeine interferes with hepatic lipid metabolism by observing a decrease in hepatic mRNA expression of fatty acid synthase and acetyl-CoA carboxylase along with an increase in hepatic carnitine palmitoyltransferase 1 (CPT1) and proliferation-activated receptor α, which suggested that the administration of caffeine regulates hepatic de novo lipogenesis and β-oxidation [73].

*Caffeine, adenosine receptors, and sport*

Caffeine is an ergogenic compound that was included on the list of prohibited substances by the World Anti-Doping Agency between 1984 and 2004, followed by its inclusion in the list of substances monitored exclusively during competitions. In fact, the consumption of caffeine by athletes has also increased after its removal from the list of prohibited substances in sports [74]. The ergogenic effects are primarily due to the antagonism of A1 receptors [75], which are dose dependent and also correlated with the increase in cAMP levels. It should be stated that the increase in cAMP is the consequence of both the blocking of A1 receptors (in low doses) and the inhibition of PDEs (in high doses) [8]. Caffeine stimulates lipolysis, following the increase in cAMP, increasing the availability of free fatty acids that are then oxidized for energy without significantly altering carbohydrate metabolism, although a glycogen-sparing effect has been observed [76].

In addition to its ergogenic effect, caffeine has been shown to have many other advantages in sports.

In an animal model, intense exercise led to increased levels of AST and creatine kinase (CK) as well as SOD and GPx activity in hepatocytes. Caffeine consumption has been shown to prevent the increase of AST, even though it does not influence the plasma CK level while decreasing the activity of oxidative enzymes [77]. It has also been suggested that moderate consumption of caffeine enhances the beneficial effects of low-intensity physical exercise, an effect demonstrated by a decrease in the level of inflammatory markers (IL-1β, IL-6, TNF-α, and interferon-gamma—INF-γ) [78].

In humans, an improvement in psychomotor state and oxidative stress markers (GPX, SOD) has been observed following the ingestion of 5 mg/kg caffeine in athletes participating in an endurance race after 26 h of sleep deprivation [79].

#### 2.3.2. Caffeine and Glutamatergic Neurotransmission

The antagonism of purinergic receptors has much more complex consequences in the context of OS modulation, the literature describing the interrelationships between caffeine administration, glutamatergic neurotransmission, and its influence on the processes involved in neurodegeneration (generation of free radicals, neuroinflammation). Thus, studies in the literature directly link the administration of caffeine and the increased level of glutamate (Glu) [80]. Animal models using caffeine have shown that caffeine increases available Glu in the nucleus accumbens (NAc) and posterior hypothalamus, to some extent, by blocking A1 receptors [80,81,82]. Because caffeine exhibits the same psychostimulant properties as other substances with an addictive effect, caffeine can have similar effects to those produced by Glu as a generator of excitotoxicity. Glu generates ROS by opening Ca^2+^ channels mediated by excessive stimulation of N-methyl-D-aspartate (NMDA) receptors, resulting in OS, oxidative damage, and apoptosis [83,84]. Another way in which caffeine is able to influence the extracellular level of Glu (by modulating glutamate/aspartate uptake) is represented by blocking A2 receptors [85]. Regarding the A2A receptor subtype, it is widely distributed in microglia and has the role of potentiating inflammatory effects [86]. Associated with this mechanism, we can also mention the negative influence on the recapture of excess Glu by glutamate transporter 1, but also the increase in the activity of phospho-extracellular signal-regulated kinases (pERK 1/2), which has an immediate effect, including the marked increase in the extracellular level of Glu, activation of microglia, and initiation of neuroinflammatory processes [86,87,88]. One interesting thing about the effects of adenosine is that it foregrounds inflammation. Thus, if in peripheral tissues adenosine mediates anti-inflammatory effects through A2A receptors, in the case of neuroinflammation, the activation of these receptors is responsible for neuronal degradation [89] through the influence exerted on glial cells, which involves the stimulation of cyclooxygenase type 2 (COX2), nitric oxide synthase (NOS), the release of prostaglandins, cytokines, and stimulation of Glu efflux from the presynaptic level [90,91,92].

In contrast, a study by Ning et al. demonstrated the protective effect of caffeine on inflammatory processes and the decrease in Glu levels in the case of acute injuries [93]. In PD investigation, multiple preclinical studies have claimed that prior exposure (for different periods of time, from one week to three weeks) of experimental animals (mice, rats) to caffeine reduced the loss of dopaminergic neuronal mass in the striatal area in a dose-dependent manner (30 mg/kg, 10 mg/kg 1 g/L in drinking water), thus conferring the neuroprotective effect [94,95]. This effect of caffeine is due to blocking A2A receptors and reducing Glu release as a result of 1-methyl-4-phenyl-1,2,3,6-tetrahydropyridine (MPTP) administration. Thus, the binding and automatic blocking of this receptor subtype reduces the activity of protein kinase A (PKA) by decreasing the level of extracellular Ca^2+^ that can penetrate inside the cell, thus lowering the amount of Glu released, hence reducing neuroinflammation [87,96].

## 3. From Pure Caffeine to Coffee Products

All the above studies have assessed the antioxidant potential of pure caffeine, which raises the question of whether the active compound from food has the same properties. The phytochemical complexity of natural extracts can influence the effect of caffeine, but we must bear in mind that foods also contain sugars and sweeteners.

Many energy drinks combine taurine with caffeine. Although it is a compound that is endogenously synthesized in the body, antioxidant effects are only visible in higher concentrations. The additional intake could bring benefits correlated with the antioxidant action (stabilization of cell membranes, inhibition of enzymes involved in the generation of ROS, modulation of transcription factors such as Nrf2 and NF-kB) [97].

### 3.1. The Importance of the Phytocomplex in the Modulation of Oxidative Stress

The main natural caffeine sources are Coffeae semen, Theae folium, Colae semen, Mate folium, Guarana, and small quantities are found in Cacao semen. The content of caffeine in coffee beans is influenced by multiple factors (genetic, environmental, agricultural, etc.) but is usually between 1–1.2% in Coffea arabica and 1.7–2.5% in Coffea canephora [98]. Xanthine alkaloids are usually found in herbal drugs complexed with different compounds such as tannins or chlorogenic acid (CGA) and caffeine chlorogenate. These polyphenolic compounds have high antioxidant capacities, and, overall, they greatly influence the systemic effects of the total extracts/beverages. Of the compounds that are extracted together with caffeine from plant products, the most important are likely catechin derivatives found in green tea. Catechins are a group of flavan-3-ols with multiple phenoxy groups, a chemical characteristic that enhances its antioxidant potential compared to other flavonoids. The most important catechin found in green tea is (−)-epigallocatechin gallate, but small quantities of (+) catechin, (−)-epicatechin, gallocatechin, gallocatechin gallate, and epicatechin gallate have been identified. In green coffee seeds, the amount of CGAs varies significantly between different Coffea species, reaching almost 15% in Coffea canephora [99]. CGA content in coffee is closely correlated with caffeine content, and the major CGA encountered is 5-O-caffeoquinolinic acid.

Often, in weight loss supplements or those intended for athletes, the actual caffeine content is higher than that stated on the label (besides the declared pure caffeine, the extracts of tea or green coffee are highlighted separately, and the total alkaloid content is higher) [100].

The antioxidant effect of CGAs has been assessed in both in vitro and in vivo studies.

For in vitro studies, CGAs have been shown to have the ability to trap free radicals [101,102,103,104] and to decrease MDA levels [105] in a dose-dependent manner, with consumption being likely to have beneficial effects for the body, including the progression of neurodegenerative diseases induced by OS. However, in addition to the direct action of neutralizing free radicals, the antioxidant effect of CGA can also be explained by the fact that it activates Nrf2, thus stimulating the activity of endogenous antioxidant enzymes [104]. In this case, as well, studies have been conducted on pure substances. However, a study assessed the ability of coffee extracts to inhibit the aggregation of pathogenic Aβ, tau fibrillization, and tau aggregation, noting that concentrated extracts, respectively dark roast coffee extract, had a significantly higher effect compared to light roast coffee extract and decaffeinated dark roast coffee, in the case of the last two types of extracts the inhibition potency of the oligomerization of Aβ plates being almost identical. In addition, pure caffeine did not have the ability to inhibit the oligomerization process. Of the compounds studied by Mancini et al. that had a favorable effect in inhibiting the aggregation of pathogenic Aβ was quercetin, while phenylidane had a beneficial effect in reducing the aggregation of both tau protein and Aβ. Chlorogenic acid did not influence these processes. Moreover, in high concentrations, caffeic acid and quercetin stimulated the aggregation of α-synuclein, a protein with implications for the pathogenesis of PD [106]. Similar results were obtained by Baeza et al., who demonstrated that pure caffeine, unlike CGAs, does not provide significant protection against OS [107].

Unfortunately, the results obtained in vitro, although they may provide information on the effect of certain substances, must be confirmed in vivo. If we refer to CGA and caffeic acid, although they are absorbed from the digestive tract, they do not cross the blood-brain barrier (BBB) [108]; therefore, the neuroprotective action of coffee extracts cannot be attributed to these compounds. Since the antioxidant action of CGA should not be neglected despite its low bioavailability, attempts have been made to obtain a CGA-loaded liposome to increase its bioavailability and antioxidant capacity in vivo [109]. Although this formulation improves the transmembrane transfer of the substance, it is not possible to benefit from such complexes in food.

The beneficial effects of caffeine explained on the basis of the antioxidant properties of the phytocomplex, have also been demonstrated in patients with hepatocellular carcinoma, with a reduced risk (in most studies) in the case of coffee-consuming patients [110,111].

Important amounts of caffeine can be found in cocoa and cocoa products. As recently reported, commercial cocoa contains, on average, 0.21% caffeine, sweet chocolate 0.07%, and milk chocolate 0.02% caffeine [112] The consumption of these products has also been correlated with beneficial effects on health, explained by a capacity to combat the effects of OS. Yet again, this is a case of a complex composition of these extracts containing increased amounts of polyphenols as well as theobromine, the main compound with a xanthine structure. Since the antioxidant capacity of products depends on the concentration of these active compounds, Belščak et al. demonstrated that the highest antioxidant protection is attributed to cocoa products containing the highest amount of solid cocoa (e.g., dark chocolate) [113].

The polyphenols contained in cocoa and green tea, especially quercetin, have a well-documented antioxidant action [114]. Even though in vitro studies have shown that quercetin crosses the BBB [115], in vivo studies in rats and pigs have shown that both quercetin and its metabolites are distributed in most internal organs, including the brain (even if in a smaller amount), provided that the animals benefit from a diet high in quercetin for a long period of time; otherwise, they are distributed only in the kidneys and liver [116]. As in the case of CGAs, the antioxidant action of quercetin is attributed to the ability to neutralize ROS, respectively RNS [117], but also by activating the Nrf2-pathway [118,119].

### 3.2. Influence of Taste Regulators on Oxidative Stress (Sugar and Sweeteners)

For understandable health reasons, the World Health Organization (WHO) recommends that the amount of free sugar intake is below the limit of 5–10% of the total energy consumed in a day [120]. This reduction in carbohydrate consumption is based on the desire to limit the conditions associated with metabolic syndrome (type 2 diabetes, endocrine diseases, ischemic heart disease, coronary heart disease, myocardial infarction) [121,122,123]. In addition, a study by Brown et al. states that each extra-sweetened drink negatively influences blood pressure values, and thus, the systolic arterial pressure increases by 1.6 mmHg, and the diastolic increases by 0.8 mmHg [124].

Since food products contain sugar or sweeteners, the dilemma is whether they might generate ROS and interfere with the antioxidant action of caffeine or phytocomplex.

First, an increase in glucose metabolism results in the production of ROS via mitochondrial glucose oxidation, with the formation of $O_{2}^{*-}$ and H_2_O_2_. Moreover, it has been observed that in the case of hyperglycemic status, the mitochondrial fission process correlates with the increase in the levels of ROS of mitochondrial origin [125]. Another pathway from which ROS results is represented by the nicotinamide adenindinucleotid phosphate oxidase system (NADPH-oxidase), which can be found in the vascular endothelium, smooth muscle cells, cardiomyocytes, and the immune system (neutrophils and macrophages). Thus, the hyperglycemic status favors the activation of NADPH-oxidase and the generation of ROS [126,127,128].

At the same time, constantly increased glucose levels promote the formation of advanced glycation end products (AGEs). These AGEs arise as a result of a non-enzymatic reaction between sugars and amino groups on proteins, lipids, or nucleic acids. These are compounds of interest due to their pathological potential and ability to generate ROS [129]. Three mechanisms for the formation of AGEs are currently known.

The first described mechanism is the Millard reaction in which the aldehyde group on the glucose attaches to the free amino group on the proteins with the formation of a Schiff base, which, after rearrangement, leads to a stable Amadori compound.

The second mechanism consists of the self-oxidation of glucose along with lipid peroxidation, which leads to the formation of dicarbonyl derivatives. These derivatives (glyoxal, methylglyoxal, and 3-deoxyglucosone) ultimately cause the formation of AGEs [130].

The third mechanism is the polyol pathway. This last mechanism involves the reduction of glucose by the action of aldose-reductase to sorbitol, which is further converted to fructose by the action of sorbitol dehydrogenase. Subsequently, fructose-3-phosphate is transformed into α-oxaldehyde, a compound that has the ability to lead to the formation of AGEs [131].

Once formed, these AGEs can react with the receptor for advanced glycation end products (RAGE), leading to the activation of the nuclear factor kappa-B (NF-κβ). Once activated, NF-κβ stimulates the transcription of cytokines (IL-1, IL-6, IL-8, and TNF-α), but also of NADPH-oxidase, all of which favor the production of ROS. The third mechanism is the polyol pathway. This last mechanism involves the reduction of glucose by the action of aldose-reductase to sorbitol, which is further converted to fructose by the action of sorbitol dehydrogenase. Subsequently, fructose-3-phosphate is transformed into α-oxaldehyde, a compound that has the ability to lead to the formation of AGEs [132,133,134,135]. Additionally, the level of AGEs correlates with the level of high-sensitivity C-reactive protein (hs-CRP), which is known to stimulate the production of ROS by neutrophils [136]. At the same time, another ROS-generating mechanism is suggested, this time via insulin, released from the massive amounts of ingested glucose. Insulin activates the type 4 isoform of NADPH-oxidase through which H_2_O_2_ is formed [137,138].

Aspartame is included in this category of sweeteners. It is added to various foods, sweets, beverages, oral hygiene products (chewing gum, breath mints), and even in pharmaceutical products as a result of their advantageous hypocaloric properties [139]. The main metabolites of aspartame are represented by approximately 50% phenylalanine, 40% aspartic acid, and 10% methanol [140]. Regarding the consumption of products containing aspartame, various neurological effects have been reported, including effects such as headaches, insomnia, and even seizures, along with changes in the level of catecholamines. In animal studies, the use of high doses of aspartame was also accompanied by neurochemical changes, with the toxicity being attributed to its pro-oxidant effect. Thus, the ROS generated by this sweetener induced an imbalance between pro-apoptotic (Bax) and anti-apoptotic markers (Bcl-2) in favor of pro-apoptotic ones [141]. During metabolism, aspartame releases methanol, which is then subjected to metabolism via three enzyme systems: alcohol dehydrogenase, catalase, and the microsomal oxidizing systems. The latter is considered directly responsible for the generation of ROS [142,143]. Methanol is also oxidized to formaldehyde which then transforms into formic acid. In addition, a close connection between methanol and corticosteroid levels is observed, as well as the presence of mitochondrial lesions, hence the increased production of ROS in conjunction with the alteration of the antioxidant mechanisms [144].

A decrease in GSH was also observed, which is correlated with increased levels of MDA generated from lipid peroxidation. This decrease in GSH is most likely due to methanol metabolites. In fact, multiple studies have demonstrated this oxidative damage, both at the hepatocellular level and in the kidneys, but, most likely, there are oxidative lesions at the central level as well [142,145,146,147,148,149]. In the same manner, a reduction in protein thiols was observed, which resulted from the oxidation of thiolic groups (-SH), which was the result of the decrease in GSH [142,145]. Another important effect to consider is that of the oxidation of -SH groups. There is a risk of a loss of ionic balance, given that ATP-ases are linked to the membrane by these groups and are susceptible to the action of ROS, being recognized as a phenomenon encountered in cytotoxicity [150,151].

Similar data in direct connection with OS were also observed in the case of other sweeteners such as saccharin, sucralose, and cyclamate, with a similar effect on the level of peroxidized lipids, GSH, but also on 8-hydroxy-20-deoxyguanosine (8-OHdG), without describing a clear mechanism of ROS generation [152,153,154,155].

However, the existence of sweetener alternatives without caloric value and with antioxidant properties should also be mentioned, such as Steviol glycosides or Siraitia grosvenorii fruit extract that have been demonstrated in the literature [156,157,158,159,160].

In conclusion, it is considered that the acceleration of the oxidative processes is due to the methanol obtained from aspartame, and the most important matter is that the most affected are diabetic patients, who have differently affected functions, including the antioxidant capacity.

## 4. Conclusions

The effects of caffeine on the CNS and the body systems are extremely complex in usual doses, explained by the antagonism of adenosine receptors. The blockade of these receptors has been associated with the potential antioxidant action of caffeine, but many of the effects may be the consequence of influencing intracellular transduction mechanisms, a certain effect based on only one of the two mechanisms being very difficult to explain. As far as the antioxidant action of pure caffeine is concerned, the properties of caffeine are intensively studied, and it has been shown to have beneficial effects on health (e.g., neurodegenerative disorders, liver damage, and physical activity). However, these results cannot be fully extrapolated in cases in which the intake of caffeine comes from products intended for human consumption, such as coffee, green/black tea, and cocoa products (sweets and beverages), for two reasons. First, these products also contain other compounds that contribute to the antioxidant capacity, in particular by the content of polyphenolic compounds, namely chlorogenic acids and quercetin. Of these, studies have shown that only quercetin, also in high doses (repeated exposure), reaches sufficient concentrations in the CNS to explain the neuroprotective effects, not only the peripheral antioxidant ones. Moreover, in the case of coffee, the degree of roasting of the beans is also important. Second, sugar and artificial sweeteners can antagonize the antioxidant action of caffeine due to their ability to induce ROS generation. However, natural sweeteners Steviol glycosides or Siraitia grosvenorii fruit extract also have antioxidant properties. It is, therefore, difficult to assess to what extent food products containing caffeine modulate the oxidative status of differences depending on the source of caffeine and the dose ingested, despite the fact that epidemiological studies report a strong link between the consumption of coffee/caffeine and lowering the risk/incidence of neurodegenerative diseases.

## Figures and Tables

**Table 1:** The proposed neuroprotective mechanisms of caffeine after in-vivo animal exposure to neurotoxic compounds.

Neurotoxic Compound	Animal Species	Caffeine Dose (mg/kgbw)	Proposed Mechanisms	Consequences	References
d-galactose	Rats	3	↓ COX-2, NOS-2, TNF-α, and IL-1β	Reduction of OS-mediated neuroinflammation and cognitive dysfunction.	[52]
AlCl_3_	Rats	1.5	↑ BDNF ↑ Nrf2	Reduction of OS-mediated neuroinflammation and apoptotic neuronal cell loss.	[56]
LPS	Mice	3	↑ Nrf2/HO-1 ↑ TRL4	Reduction of OS-mediated neuroinflammation and synaptic dysregulation.	[54]
LPS	Mice	40	Modulation of glutamatergic neurotrasmition	Reduction of activated microglia and OS-mediated neuroinflammation.	[57]
Cd	Mice	30	↑ Nrf2/HO-1	Reduction of OS-mediated neuroinflammation and cognitive dysfunction.	[53]
MPTP	Mice	10	Modulation of glutamatergic neurotrasmition	Neuroprotection.	[58]
6-HODA	Rats	10 or 20	↓ TNF-α/IL-1β	Neuroprotection.	[59]

COX-2 (cyclo-oxygenase-2), NOS-2 (nitric oxide synthase-2), OS (oxidative stress), TNF-α (tumor necrosis factor-α), IL-1β (interleukin-1β), BDNF (brain-derived neurotrophic factor), Nrf2 (nuclear erythroid 2-related factor), HO-1 (heme oxygenase-1), LPS (lipopolysaccharides), TLR 4 (Toll-like receptor 4), MPTP (1-methyl-4-phenyl-1,2,3,6 tetra-hydropyridine), 6HODA (6 hydroxydopamine).

**Table 2:** The association of caffeine/coffee intake with the risk of neurodegenerative disorders.

Study Type	Caffeine/Coffee Ingestion	The Study’s Conclusions	References
Epidemiological (21-year follow-up)	Coffee	Lower risk of dementia and AD	[63]
Epidemiological (30-year follow-up)	Caffeine/coffee	Lower incidence of PD	[64]
Epidemiological (22-year follow-up)	Caffeine/coffee	Reduced risk of PD	[65]
Epidemiological (10-year follow-up for men 16-year follow-up for women)	Caffeine/coffee	Protective effect on risk of PD	[66]
Systematic review and meta-analysis	Caffeine/coffee	Inverse association between caffeine intake and risk of PD	[67]
Meta-analysis	Caffeine/coffee	Caffeine modified disease risk and progression in PD	[68]

PD (Parkinson’s diseases), AD (Alzheimer’s diseases).

## Data Availability

Not applicable.
